# Spatio-temporal characterization of *Trypanosoma cruzi* infection and discrete typing units infecting hosts and vectors from non-domestic foci of Chile

**DOI:** 10.1371/journal.pntd.0007170

**Published:** 2019-02-15

**Authors:** Camila Ihle-Soto, Eduardo Costoya, Juana P. Correa, Antonella Bacigalupo, Berenice Cornejo-Villar, Viviana Estadella, Aldo Solari, Sylvia Ortiz, Héctor J. Hernández, Carezza Botto-Mahan, David E. Gorla, Pedro E. Cattan

**Affiliations:** 1 Facultad de Ciencias Veterinarias y Pecuarias, Universidad de Chile, Santiago, Región Metropolitana, Chile; 2 Facultad de Ciencias, Universidad de Chile, Santiago, Región Metropolitana, Chile; 3 Instituto de Ciencias Biomédicas, Facultad de Medicina, Universidad de Chile, Santiago, Región Metropolitana, Chile; 4 Facultad de Ciencias Forestales y de la Conservación de la Naturaleza, Universidad de Chile, Santiago, Región Metropolitana, Chile; 5 Instituto de Diversidad y Ecología Animal, CONICET, Universidad Nacional de Córdoba, Córdoba, Argentina; Universidad de Buenos Aires, ARGENTINA

## Abstract

**Background:**

*Trypanosoma cruzi* is a protozoan parasite that is transmitted by triatomine vectors to mammals. It is classified in six discrete typing units (DTUs). In Chile, domestic vectorial transmission has been interrupted; however, the parasite is maintained in non-domestic foci. The aim of this study was to describe *T*. *cruzi* infection and DTU composition in mammals and triatomines from several non-domestic populations of North-Central Chile and to evaluate their spatio-temporal variations.

**Methodology/Principal findings:**

A total of 710 small mammals and 1140 triatomines captured in six localities during two study periods (summer/winter) of the same year were analyzed by conventional PCR to detect kDNA of *T*. *cruzi*. Positive samples were DNA blotted and hybridized with specific probes for detection of DTUs TcI, TcII, TcV, and TcVI. Infection status was modeled, and cluster analysis was performed in each locality. We detected 30.1% of overall infection in small mammals and 34.1% in triatomines, with higher rates in synanthropic mammals and in *M*. *spinolai*. We identified infecting DTUs in 45 mammals and 110 triatomines, present more commonly as single infections; the most frequent DTU detected was TcI. Differences in infection rates among species, localities and study periods were detected in small mammals, and between triatomine species; temporally, infection presented opposite patterns between mammals and triatomines. Infection clustering was frequent in vectors, and one locality exhibited half of the 21 clusters found.

**Conclusions/Significance:**

We determined *T*. *cruzi* infection in natural host and vector populations simultaneously in a spatially widespread manner during two study periods. All captured species presented *T*. *cruzi* infection, showing spatial and temporal variations. *Trypanosoma cruzi* distribution can be clustered in space and time. These clusters may represent different spatial and temporal risks of transmission.

## Author summary

*Trypanosoma cruzi* is a parasite that infects mammals, transmitted by triatomine insect vectors in America, causing Chagas disease in humans. There are six *T*. *cruzi* discrete typing units (DTUs). Our goal was to estimate *T*. *cruzi* infection rates and describe the DTUs present in mammals and triatomines of Chile, evaluating spatial and temporal variation. We captured nine small mammal and two triatomine species in six localities during two periods (summer/winter) of the same year. We detected *T*. *cruzi* DNA and some DTUs were identified. We report one mammal species infected for the first time. Infection presented significant variation among species. The endemic vector had higher infection rates than *Triatoma infestans*. The DTUs TcI, TcII, TcV and TcVI were present, with predominance of TcI. Temporally, we detected higher rates of infection during summer in small mammals and during winter in triatomines. Infection was spatially and temporally aggregated in small mammals and vectors. Some species might have higher risk of infection, and this may be different between localities or periods, or even within the same locality.

## Introduction

Chagas disease is a zoonotic parasitic disease, endemic in 22 countries of America, caused by the flagellated protozoa *Trypanosoma cruzi*. This disease affects approximately 7 million people in the world and represents the third parasitic disease of major world impact [1]. The parasite is transmitted through contact of contaminated feces of hematophagous insects from the Triatominae subfamily with wounds or mucosae of mammals, by blood transfusions, congenital transmission, organ transplants, laboratory accidents, and oral transmission [2]. Vectorial transmission occurs from southern United States to Patagonia (40°N to 45°S) [3, 4]; however, in the last decades, Chagas disease has spread to other continents due to alternative infection routes and migration [1]. In Chile, the disease is endemic in rural and suburban areas from latitudes 18°30’ to 34°36’ S [5].

*Trypanosoma cruzi* is a mono-flagellar protist (Kinetoplastida). Its kinetoplastidic DNA (kDNA) displays like a concatenated discal web of maxicircles (20–40 kb; 20–25 copies/cell) and minicircles (0.5–10 kb; 20000 copies/cell) [6]. Minicircles are organized in four conservative regions (conserved sequence blocks, CSB) separated by four variable regions [7]. Due to the high number of minicircle copies and conserved sequences, they are used in the diagnosis of infection through polymerase chain reaction (PCR), using primers that bind to the CSB [8]. Minicircle DNA amplification creates a very polymorphic product of the variable region, useful for *T*. *cruzi* genotyping through hybridization methods using characterized probes [9]. Six genetically related lineages of *T*. *cruzi* have been described, identifiable by markers, called discrete typing units (DTUs): TcI, TcII, TcIII, TcIV, TcV, TcVI [10]. A new genotype called TcBat has been discovered in bats from Brazil [11]. Several approaches have been used to evaluate the biochemical and genetic diversity of *T*. *cruzi* isolates, but there is no unique genetic target that allows complete DTU resolution [12].

TcI exhibits the wider distribution, from Southern USA to Central Chile and Argentina [3]. TcII is found primarily in the domestic cycle from the South-Central region of South America. TcIII ranges from western Venezuela to the Argentine Chaco, mainly linked to the wild cycle in Brazil [13]. TcIV possess a similar distribution to TcIII but is absent in the Gran Chaco area. Finally, TcV and TcVI are found in Central and Southern South America [12]. So far, no clear association between the parasite genotype and the manifestation of the disease or drug resistance has been detected [14], but there is evidence that suggests a selective role of hosts and vectors on the different DTUs [15–17].

More than 150 wild, synanthropic, and domestic mammal species have been found infected with *T*. *cruzi* in America, including most of the terrestrial mammal orders present [3, 18], playing a relevant role in the maintenance and interplay among wild, peridomestic and domestic cycles [19]. Small mammals are common feeding sources for triatomines in the sylvatic cycle of the endemic zone of Chile [20], presenting smaller home ranges than larger mammal species [21]. Since the home range of triatomines is also small [22], these mammals can act as important *T*. *cruzi* hosts, acquiring and maintaining the infection [23, 24]. Infected species in Chile are the rodents *Octodon degus*, *Phyllotis darwini*, *Abrothrix olivaceus*, *Rattus rattus*, the lagomorph *Oryctolagus cuniculus*, and the marsupial *Thylamys elegans*, ranging from 32% to 83.6% [18, 23, 25–27]. North-Central Chile is a Mediterranean climatic influenced area characterized by lower richness of terrestrial mammals than other Mediterranean areas of the world [28], and over 40% of the 30 wild or synanthropic mammal species present are small mammals, exhibiting relatively high abundances [29].

Triatomines can get infected with *T*. *cruzi* at any stage posterior to hatching, by consumption of contaminated mammal blood, cannibalism or coprophagy [3]. In Chile there are four triatomine species: *Triatoma infestans*, *Mepraia spinolai*, *M*. *parapatrica*, and *M*. *gajardoi* [30, 31], where *T*. *infestans* has been found in domiciliary and wild habitats [32, 33], while *M*. *spinolai* in domestic, peridomestic but mainly wild habitats [34]. *Mepraia gajardoi* and *M*. *parapatrica* are present in wild coastal areas [31]. *Mepraia spinolai* and *T*. *infestans* are distributed sympatrically in part of the endemic area [35]. Infection rates of *T*. *cruzi* detected by conventional PCR in sylvatic *T*. *infestans* and *M*. *spinolai* from Chile vary spatially and temporally, ranging from 36.5% to 68.6% and 14.9% to 76.1%, respectively [32, 33, 36–38]. TcI is the most frequently circulating DTU in *T*. *infestans* [36], and *M*. *spinolai* [37], as well as in Chilean small mammals, present as single and mixed infections [16, 27]. However, there are differences between species regarding their infecting DTU [16, 25, 27, 36, 37].

Infection events can have one of three different spatial configurations: regular or uniform, random, or aggregated (clustered); however, to our knowledge the spatial configuration of *T*. *cruzi* infection in hosts and vectors has not been previously evaluated. To understand transmission cycles, it is important to establish whether cases of an infection–i.e., the infected individuals—have the tendency to cluster together more than it would be expected by the natural clustering of the population affected [39]. In the present study, we aimed to assess spatial and temporal variations of *T*. *cruzi* infection, detecting the DTUs, by sampling triatomines and small mammals of the same areas in two contrasting seasons of the same year, using conventional and spatially-explicit statistical techniques.

## Methods

### Trapping sites and dates

Small mammals and triatomines were captured from January to February (austral summer season) and from July to August (austral winter season) of 2011. The six study sites—Localities 1 to 6—were in North-Central Chile, from 30º49’S to 33º39’S, encompassing around 300 km from the northernmost to the southernmost study site (S1 Fig). Most of the rainfall in all study sites concentrates between May and August, which are also the colder months [40] Details of each locality are shown in S1 Table. Base layers (shapefiles) of administrative boundaries, rivers and elevation were obtained from freely available sources for academic use and other non-commercial use [41, 42]; point shapefiles of trapping sites and maps were produced specifically for this investigation, in QGIS Desktop 2.18.2 software, a free and open source Geographic Information System [43].

### Small mammal sampling

Small mammals were captured using live traps (Rodentrap Special Forma and Rodentrap Berlin Forma, Santiago, Chile, and Tomahawk traps, Wisconsin, USA) with rolled oat as bait and cotton as shelter for the captured animals. Traps were placed in linear patterns separated by approximately 10 m, labeled and georeferenced. Each captured mammal was anesthetized with isoflurane and blood sampled in a field laboratory. Detailed procedure is available at dx.doi.org/10.17504/protocols.io.wnxfdfn.

#### Ethics statement

Sampling procedures were authorized by the Servicio Agrícola y Ganadero (SAG Resolution N° 6853) and by the Bioethics Committee of the Facultad de Ciencias Veterinarias y Pecuarias, Universidad de Chile (Certificate Nº 17), regulated in Chile according to Ley 19.473 about wildlife hunt and capture [44], and Ley 20.380 about animal protection [45]. Live capture, marking, holding, maintenance, and blood withdrawal followed international guidelines for wild and laboratory mammals [46, 47].

### Triatomine sampling

Triatomines were captured using baited traps. A total of 90 yeast baited traps per day were set during summer, and 72 mouse baited traps during winter. Traps were placed following linear patterns, separated by 10 m in rocky outcrops and rock piles; and assorted according to the availability of terrestrial bromeliads if present. Each trap was georeferenced in UTM WGS84 19S coordinate system. Traps were activated at sunset and collected the next morning. Detailed capture procedure is available at dx.doi.org/10.17504/protocols.io.wnpfddn. Captured triatomines were transferred to individual flasks for transportation. Triatomine species were identified based on its morphological description [30, 48]. Insects were euthanized with ether overdose, and their abdomens were dissected using individual scalpels.

### DNA extraction

DNA was extracted from small mammals’ blood samples (100 μl) using the Quick-gDNA Blood MiniPrep kit. Triatomines’ abdomens were macerated with 190 μl Guanidine-HCl 6 M—EDTA 0.2 M solution and incubated with 10 μl of proteinase K solution (20 mg/ml) during 3 hours at 54°C. Samples were then centrifuged for 1 min at 10.000 x *g*; the supernatant was transferred to another microcentrifuge tube and its DNA was extracted using the Quick-gDNA MiniPrep (Zymo Research) kit. Both blood and triatomine eluates were resuspended in 100 μl nuclease free water.

### *Trypanosoma cruzi* infection

*Trypanosoma cruzi* infection status of each DNA sample was determined by conventional PCR using a master mix containing 5 μl of DNA sample, buffer solution 1x; dATP, dCTP, dGTP y dTTP 0.38 mM each; MgCl_2_ 1.37 mM; 1.3 units of *Paq* DNA Polimerase (Agilent); 0.4 μM of each oligonucleotide: 121 and 122, which anneal to CSB2 and CSB3 of *T*. *cruzi*’s kinetoplast minicircles, respectively [49]; and nuclease free water to complete 32 μl. Each run included a positive (purified *T*. *cruzi* kDNA) and a template free control (nuclease free water). Amplification was performed with a cycling protocol of: 1 min at 98°C and 2 min at 64°C; followed by 33 cycles at: 94°C for 1 min and 64°C for 1 min; ending with a 10 min cycle at 72°C. Ten μl of amplified samples were run in a 2% Tris-Borate-EDTA agarose gel with GelRed nucleic acid stain for 60 min at 90 volts. Samples were considered positive to *T*. *cruzi* infection when a 330 pair base band was observed by ultraviolet transillumination after electrophoresis.

### Genotyping

PCR positive samples were genotyped with a DNA blot technique. In this procedure it is expected that two minicircle sequences will cross-react if hybridized under high stringency conditions only if they belong to the same sequence class; that is, if they present homologies in the divergent region [50]. Minicircle hybridization is a complex technique that has the advantage of working with low parasite amounts, and may be used for direct genotyping without the bias of parasite isolation and culture, which may favor the selection of some *T*. *cruzi* clones from a mixture [14, 51]. Further details are specified in dx.doi.org/10.17504/protocols.io.sz2ef8e.

### Statistical analysis

R software version 3.5.1, with packages rcompanion, RVAideMemoire, Lme4 and epiDisplay, were used for statistical analyses. Descriptive statistics of the infection status were included for species, localities, and study periods. Differences in the frequencies of infection were analyzed by species using Fisher’s exact test, testing *a posteriori* differences between mammal species using a pairwise test of independence for nominal data, with a significance level of α = 0.05. The infection status of vectors was modeled using the locality (1–6), study period (summer vs. winter) and the species (*T*. *infestans* and *M*. *spinolai*) as predictors in a factorial logistic regression. A separate model was generated for the infection status of small mammals, using three categories for the species variable: *Octodon* sp., *P*. *darwini*, and all other small mammals combined, along with the variables locality and study period. Bar charts were created in Microsoft Excel (Microsoft Office Professional Plus 2010, version 14.0.7208.5000).

Cluster analysis was performed using SaTScan v9.4.4 64-bit software (Kulldorff M. and Information Management Services, Inc. SaTScan v8.0: Software for the spatial and space-time scan statistics. http://www.satscan.org/, 2009. “SaTScan is a trademark of Martin Kulldorff. The SaTScan software was developed under the joint auspices of (i) Martin Kulldorff, (ii) the National Cancer Institute, and (iii) Farzad Mostashari of the New York City Department of Health and Mental Hygiene”). Spatial, temporal and space-time cluster detection were performed in each locality for small mammals and triatomines, separated and combined. We used the default software settings except by using an elliptical scanning window and 9999 iterations of Standard Monte Carlo procedure for calculations [52, 53]. To determine if there was clustering of the infection status, we used the Bernoulli model [54], where each individual (triatomine or small mammal) was either a case (1)—which corresponded to an infected vector or host—or a control (0)–an uninfected individual.

## Results

### Trapping results

**Fig 1:**
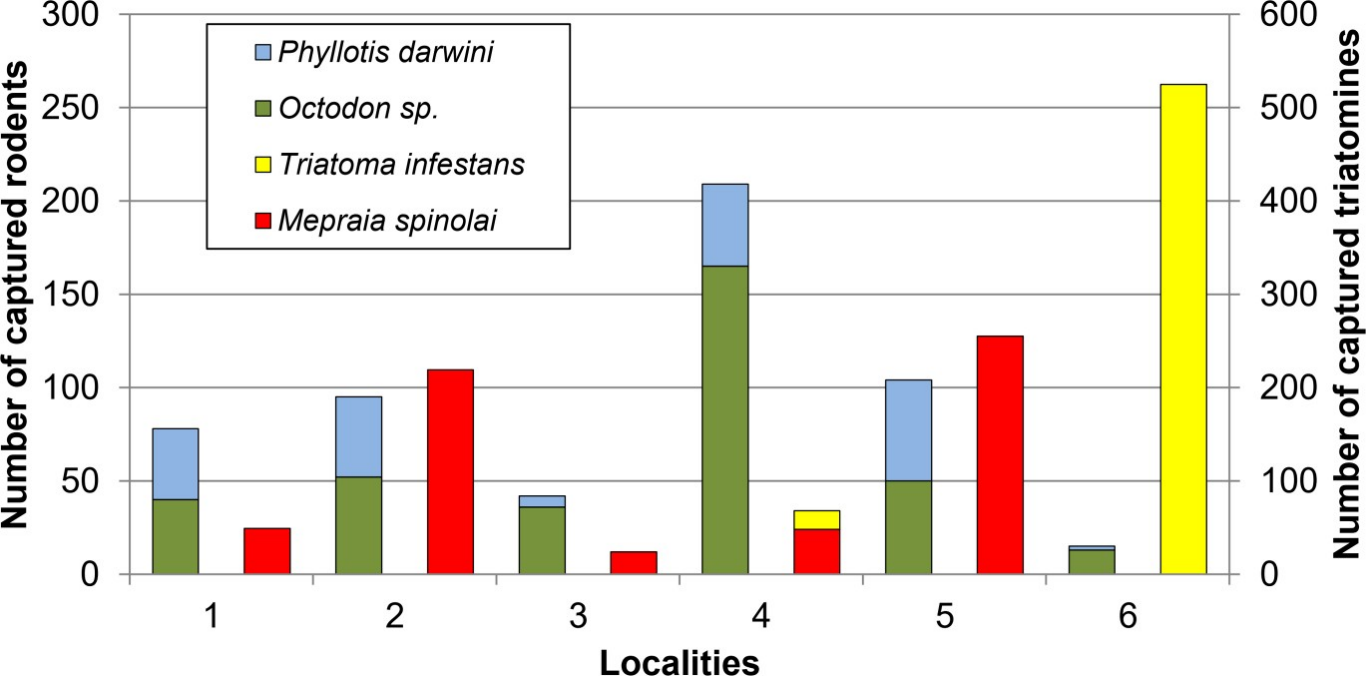
Trapping results of the most abundant small mammals and triatomines, by locality. The horizontal axis shows the Localities; the left vertical axis refers to *Octodon* sp. and *P*. *darwini*; and the right vertical axis refers to *T*. *infestans* and *M*. *spinolai*.

A total of 710 small mammals and 1140 triatomines were captured. Small mammals belonged to two rodent Suborders: Hystricomorpha and Myomorpha, and to one marsupial Order: Didelphimorphia [18]. Almost 76.5% of the captured mammals were *Octodon* sp. (n = 356) and *P*. *darwini* (n = 187). *Mepraia spinolai* (n = 595) and *T*. *infestans* (n = 545) were collected, found in sympatry only in Locality 4 (Fig 1). Detailed number of small mammals and triatomines captured by locality and study period is available in S2 Table.

### Infection status

All infection results are presented indicating the average and the 95% confidence interval, in Tables 1 and 2. We detected 215 small mammals infected with *T*. *cruzi* (30.3% of infection; 95% CI 27.0–33.7%), presenting different infection rates among mammal species, without considering locality or study period (Fisher’s exact test, p<0.001). *A posteriori* pairwise comparisons showed that only *Octodon* sp. and *P*. *darwini* were statistically different (adjusted p = 0.0287), with *P*. *darwini* showing higher rates of infection (39.0%) than *Octodon* sp. (25.0%). *Rattus norvegicus* and *A*. *longipilis* showed the highest and lowest infection rates, respectively. Locality 3 showed the highest, and Locality 1 showed the lowest infection rate when considering all mammals combined. During the summer, infection of small mammals was 35.6% and in the winter was 25.6%. In summer, the most and the less infected species were *A*. *olivaceus* and *O*. *longicaudatus*, respectively. During winter, *R*. *norvegicus* and *A*. *longipilis* presented the highest rate and lowest infection rates, respectively. Detailed results of infection are presented in Tables 1 and 2, and in S2 Table. In the factorial logistic regression, all the tested variables were retained as predictors for infection status of small mammals. *Phyllotis darwini* and other small mammals presented greater odds of infection than *Octodon* sp; Locality 1 presented lower odds than all the rest localities; finally, small mammals exhibited lower odds of being infected in winter versus summer (Table 3).

**Table 1:** *Trypanosoma cruzi* infection in small mammals and triatomines by locality and study period.

Locality		Study period				Total M or T from each Locality, both study periods combined	
		Summer		Winter			
		I %	95% CI	I %	95% CI	I % (ni/nt)	95% CI
**Mammals**	**1**	20.8	11.9–33.6	9.0	4.4–17.0	13.4 (19/142)	8.7–20.0
	**2**	47.5	36.9–58.3	11.4	4.5–24.4	34.7 (43/124)	26.9–43.4
	**3**	48.6	33.5–64.1	40.0	16.7–68.9	46.8 (22/47)	33.3–60.8
	**4**	26.2	18.7–35.3	31.2	23.7–39.8	28.9 (67/232)	23.4–35.0
	**5**	38.5	24.9–54.1	34.9	25.6–45.7	36.1 (44/122)	28.1–44.9
	**6**	53.3	30.1–75.2	42.9	26.5–61.0	46.5 (20/43)	32.5–61.1
**Triatomines**	**1**	35.9	22.7–51.6	20.0	4.6–52.1	32.7 (16/49)	21.2–46.7
	**2**	42.9	35.0–51.1	35.4	27.8–46.5	40.2 (88/219)	33.9–46.8
	**3**	23.5	9.1–47.8	57.1	25.0–84.3	33.3 (8/24)	17.8–53.4
	**4**	26.5	17.4–38.1	-	-	26.5 (18/68)	17.4–38.1
	**5**	41.1	34.3–48.2	50.8	38.9–62.5	43.5 (111/255)	37.6–49.7
	**6**	28.1	24.4–32.1	33.3	11.7–64.9	28.2 (148/525)	24.5–32.2

I % = Percentage of infection; CI = Confidence interval; ni/nt = Number of infected M or T/Total number of M or T of that locality, respectively; M = Mammals; T = Triatomines;— = No captures occurred, so I % and 95% CI could not be calculated.

**Table 2:** *Trypanosoma cruzi* infection by species and study period.

Species	Study period				Both study periods combined	
	Summer		Winter			
**Mammals**	**I %**	**95% CI**	**I %**	**95% CI**	**I % (ni/nt)**	**95% CI**
*A*. *bennetti*	28.6	7.6–64.8	0.0	-	22.2 (2/9)	5.3–55.7
*Octodon* sp.	29.9	24.0–36.5	18.4	13.0–25.4	25.0 (89/356)	20.8–29.8
*A*. *longipilis*	0.0	-	10.0	1.6–31.3	9.5 (2/21)	1.5–30.1
*A*. *olivaceus*	57.1	36.5–75.6	20.0	7.5–42.2	39.0 (16/41)	25.6–54.3
*O*. *longicaudatus*	25.0	6.3–59.9	16.2	7.3–31.5	17.8 (8/45)	9.0–31.6
*P*. *darwini*	44.6	33.2–56.7	36.1	28.1–44.9	39.0 (73/187)	32.3–46.2
*R*. *norvegicus*	50.0	9.5–90.6	80.0	36.0–98.0	71.4 (5/7)	40.1–93.7
*R*. *rattus*	52.4	32.4–71.7	33.3	11.7–64.9	46.7 (14/30)	28.4–62.5
*T*. *elegans*	0.0	-	50.0	25.4–74.6	42.9 (6/14)	21.3–67.5
**Total M**	**35.6**	**30.7–41.0**	**25.6**	**21.5–30.2**	**30.3 (215/710)**	**27.0–33.7**
**Triatomines**	**I %**	**95% CI**	**I %**	**95% CI**	**I. % (ni/nt)**	**95% C.I.**
*T*. *infestans*	28.0	24.4–31.9	33.3	11.7–64.9	28.1 (153/545)	24.5–32
*M*. *spinolai*	38.9	34.5–43.6	41.6	34.3–49.3	39.7 (236/595)	35.8–43.7
**Total T**	**32.9**	**30.0–35.9**	**41.2**	**34.1–48.7**	**34.1 (389/1140)**	**31.4–36.9**

I % = Percentage of infection; CI = Confidence interval; ni/nt = Number of infected of a species/Total number of individuals of that species; M = Mammals; T = Triatomines;— = No infected individuals present, so 95% CI could not be calculated.

**Table 3:** Factorial logistic regression models reporting odds ratio (OR), for infection status.

Variables	Coefficients Estimate	Pr(>|z|)	adj. OR(95%CI)	p(Wald´s test)	p(LR-test)
INFECTION STATUS FOR SMALL MAMMALS					
Intercept	-2.159	6.72e-13			
**Study period: winter vs summer**					<0.001
Winter	-0.6338	0.000624	0.53 (0.37–0.76)	<0.001	
**Species Category: ref. = *Octodon* sp.**					<0.001
*Phyllotis darwini*	1.0051	3.66e-06	2.73 (1.79–4.18)	<0.001	
Other small mammal species	0.7455	0.002146	2.11 (1.31–3.39)	0.002	
**Localities ref. = Locality 1**					<0.001
Locality 2	1.1876	0.000223	3.28 (1.75–6.16)	<0.001	
Locality 3	1.9563	1.38e-06	7.07 (3.2–15.65)	<0.001	
Locality 4	1.2891	2.57e-05	3.63 (1.99–6.62)	<0.001	
Locality 5	1.4368	9.54e-06	4.21 (2.23–7.95)	<0.001	
Locality 6	1.8890	3.65e-06	6.61 (2.97–14.71)	<0.001	
INFECTION STATUS FOR TRIATOMINES					
Intercept	-0.94082	<2e-16			
**Species: ref. = *Triatoma infestans***					
*Mepraia spinolai*	0.52133	0.00004	1.68 (1.31–2.16)	< 0.001	

We detected 389 triatomines infected with *T*. *cruzi* (34.1% of infection; 95% CI 31.4–36.9%), with higher infection rates in *M*. *spinolai* (39.7%) than *T*. *infestans* (28.1%) when comparing both species without considering locality or study period (Fisher's exact test, p<0.001). Locality 5 presented the highest triatomine infection rates (*M*. *spinolai*: 43.5%) and Locality 4 the lowest (both triatomine species combined: 26.5%; *M*. *spinolai*: 27.1%; *T*. *infestans*: 25.0%). Disregarding triatomine species and locality, higher infection rates were detected during winter (41.2%, n = 170) compared to summer (32.9%, n = 970). During summer, *Mepraia spinolai* presented 38.9% of infection, and *T*. *infestans* 28.0%, combining all localities, and in winter, *M*. *spinolai* showed 41.6%, and *T*. *infestans* 33.3%. During summer, Locality 2 had the highest rate of infection, and Locality 3 the lowest. Meanwhile, during winter, Locality 3 presented the highest infection rates, and Locality 1 the lowest. Detailed results of infection are presented in Tables 1 and 2, and in S2 Table. The model selected for triatomines retained only the species as predictor, showing that *M*. *spinolai* individuals were more frequently infected than *T*. *infestans* (p<0.001; Table 3).

### Genotyping

In small mammals, 45 out of 215 positive PCR samples hybridized with at least one probe tested (45 effective hybridizations). Only 110 out of 389 triatomine positive samples corresponded to effective hybridizations. We detected, in decreasing frequency, TcI, TcII, TcVI and TcV in small mammals (Table 4). Positive samples from *A*. *bennetti*, *A*. *olivaceus* and *R*. *norvegicus* did not bind to any probe. Only one positive sample of *A*. *longipilis* and *R*. *rattus* hybridized with TcI and TcII as single infections, respectively. Only *Octodon* sp. and *P*. *darwini* presented all four DTUs tested (S3 Table). The DTUs detected in triatomines were TcI, TcII, TcV and TcVI, in decreasing frequency. In *M*. *spinolai* TcV was not detected, and TcVI was detected in only one sample (Table 4).

**Table 4:** Number of individuals detected infected with each DTU, by species. One individual may be infected with more than one DTU. M = Mammals; T = Triatomines.

**Mammals**	**TcI**	**TcII**	**TcV**	**TcVI**	**Total number of individuals with DTUs detected**
***A*. *bennetti***	0	0	0	0	**0**
***Octodon* sp.**	5	5	3	2	**11**
***A*. *longipilis***	1	0	0	0	**1**
***A*. *olivaceus***	0	0	0	0	**0**
***O*. *longicaudatus***	1	0	1	1	**2**
***P*. *darwini***	21	14	7	9	**27**
***R*. *norvegicus***	0	0	0	0	**0**
***R*. *rattus***	0	1	0	0	**1**
***T*. *elegans***	3	1	0	0	**3**
**Total M with each DTU**	**31**	**21**	**11**	**12**	**45**
**Triatomines**	**TcI**	**TcII**	**TcV**	**TcVI**	**Total number of individuals with DTUs detected**
***T*. *infestans***	54	26	24	16	**66**
***M*. *spinolai***	34	17	0	1	**44**
**Total T with each DTU**	**88**	**43**	**24**	**17**	**110**
**TOTAL DTUs IN M+T COMBINED**	**119**	**64**	**35**	**29**	**155**

We detected the four DTUs in all localities, except in Localities 2 and 4 where TcV was not detected. Locality 6 was the only study site with all four DTUs detected both in triatomines and small mammals. Disregarding locality, during summer in *Octodon* sp. and *P*. *darwini* only TcI and TcII were detected, as single infections, but during winter, all four DTUs were found. We observed the opposite pattern in triatomines, in which we detected all four DTUs during summer and just TcI and TcII in winter. Detailed results of DTUs are shown in S3 Table.

In small mammals, we detected 62.2% single infections (hybridization with just one DTU) and 37.8% mixed infections (hybridization with more than one DTU) (Table 5). When analyzing the two most abundant species, *Octodon* sp. showed more single (72.7%) than mixed infections (27.3%), while *P*. *darwini* presented a similar proportion of single (55.6%) and mixed (44.0%) infections. In triatomines, we detected 66.4% of single and 33.6% mixed infections, with *T*. *infestans* showing more mixed infections than *M*. *spinolai* (43.9% v/s 18.2%, respectively) (Table 5). We detected a mixed infection in one *O*. *longicaudatus* with TcI+TcVI, and a single infection in the same rodent species with TcV. The marsupial species *T*. *elegans* presented a mixed infection with TcI+TcII, and the other two with DTU determined were single infections with TcI.

**Table 5:** Number of individuals according to type of infection (single or mixed), by species. Only effective hybridizations are included. A positive individual was categorized as having either single or mixed infections.

**Mammals**	**Single infection**	**Mixed infection**	**Total M**
*Octodon* sp.	8	3	11
*A*. *longipilis*	1	0	1
*O*. *longicaudatus*	1	1	2
*P*. *darwini*	15	12	27
*R*. *rattus*	1	0	1
*T*. *elegans*	2	1	3
**Total M**	**28**	**17**	45
**Triatomines**	**Single infection**	**Mixed infection**	**Total T**
*T*. *infestans*	37	29	66
*M*. *spinolai*	36	8	44
**Total T**	**73**	**37**	110

M: Mammals; T: Triatomines

When evaluating single and mixed infections by locality, there is not a clear pattern, but it seems that single infections were more frequent in both small mammals and triatomines. We did not detect mixed infections in small mammal species during summer. In triatomines we detected similar proportions of single and mixed infections in both study periods.

### Cluster analysis

**Fig 2:**
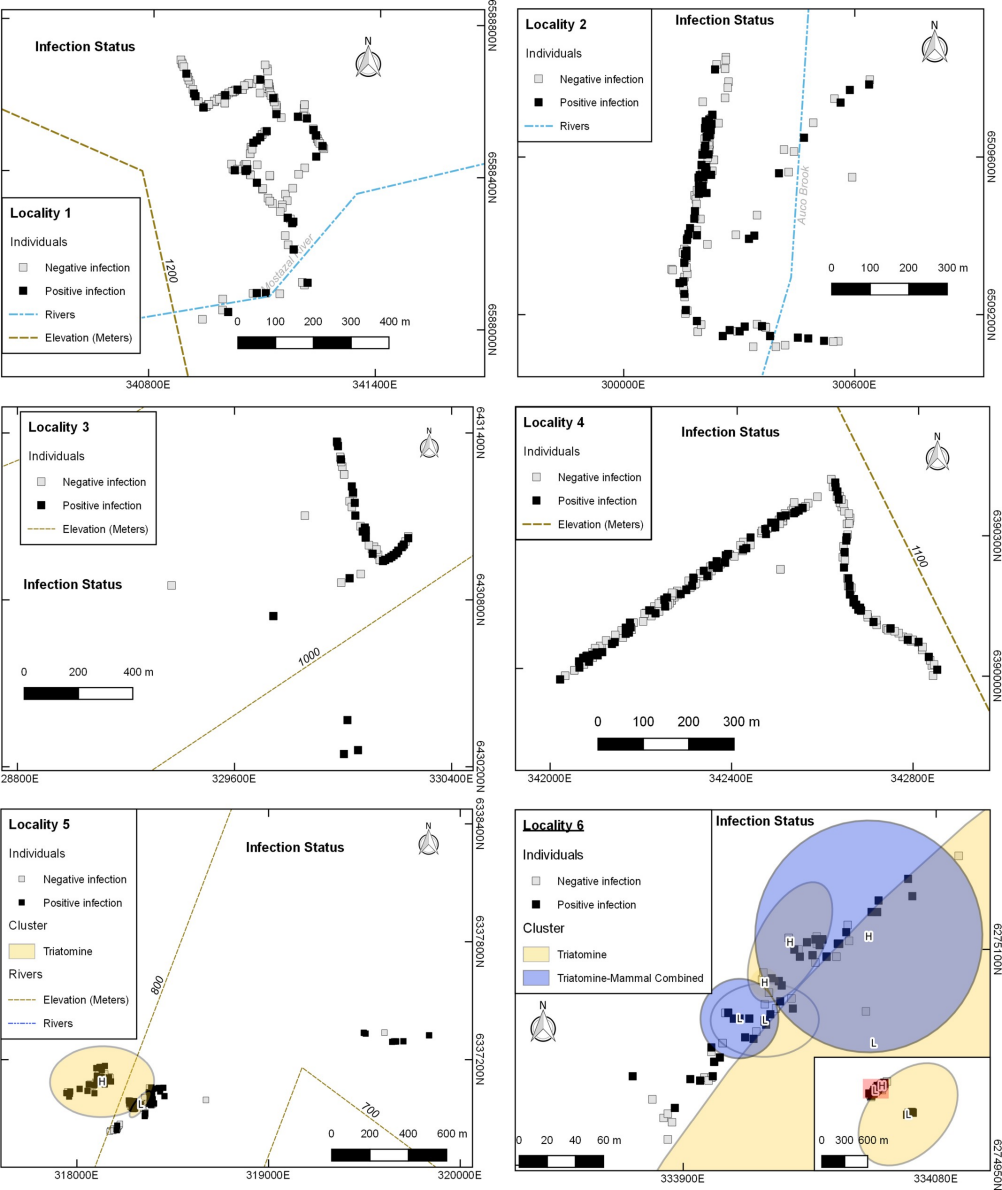
Maps with the position of the studied individuals in each Locality showing their *T*. *cruzi* infection status and the purely spatial clusters detected. H = cluster of high infection; L = cluster of low infection. Projected coordinates, UTM WGS84 19 S.

We detected a total of 21 significant spatial, temporal and spatio-temporal clusters in five localities (S4 Table). In general terms, *T*. *cruzi* clustering was more common in vectors than in hosts, with a total of 10 purely spatial and spatio-temporal clusters detected in triatomines in three localities; when combining vectors and hosts, we found 9 clusters. Most clusters were detected in Locality 6 (11 out of 21). We mapped only purely spatial clusters of infection (Fig 2).

## Discussion

In this study, we analyzed spatio-temporal infection and DTUs detected in populations of small mammals and triatomines from an endemic region of Chile. Here, the rodents *Octodon* sp. and *P*. *darwini*, and the triatomines *M*. *spinolai* and *T*. *infestans* appear to be particularly important wild hosts and vectors of *T*. *cruzi*, respectively. All tested DTUs were detected, with predominance of TcI. Infection varied among species, localities and study periods in small mammals, and between triatomines. Significant spatial, temporal and spatio-temporal clusters for infection were detected, mainly in vectors from the southernmost localities.

*Octodon* sp. and *P*. *darwini* were the most frequent and ubiquitously captured mammal species in this study. Octodontids’ specimens were not identified at species level; however, we assumed that they were *Octodon degus*. The infection rates of both rodent species might be related to their higher relative abundances and life history traits, increasing their probability to become a feeding source for triatomines due to higher contact rates [20, 55]. These two rodent species have partially overlapping distributions and home ranges [56]. *Octodon* sp. has been found associated to *Puya* sp., a terrestrial bromeliad of semiarid Chile [57], described as refuge for *T*. *infestans* and *M*. *spinolai* [32]. *Phyllotis darwini*’s nests are commonly found within abandoned *Octodon* sp. burrows [58]. Despite these species’ ecological proximity, *P*. *darwini* exhibited significantly higher infection rates than *Octodon* sp. This difference might be explained by their behavior: *P*. *darwini* is nocturnal and *M*. *spinolai* diurnal, making this host easily available for the triatomine during the day, when *P*. *darwini* rests. On the contrary, the nocturnal *T*. *infestans* would feed on the diurnal *O*. *degus* during the night [27, 33]. This different infection rate is relevant, given that infected *P*. *darwini* are reported to travel more than the uninfected, dispersing the parasite, while infected *O*. *degus* move less than uninfected specimens [59].

Synanthropic species—*R*. *norvegicus* and *R*. *rattus—*were less abundant in this study, but had high infection rates, as previously reported in Chile [23, 27]. These rodents could have an important role in Chagas disease epidemiology, since they circulate in sylvatic and domestic areas [23, 60]. Some small mammal species captured were not abundant but were nonetheless infected with *T*. *cruzi*. To our knowledge, this is the first report of infection in *O*. *longicaudatus*, with eight out of 45 infected. *Thylamys elegans*, *A*. *bennetti* and *A*. *longipilis* were also infected, with 42,9%, 22.2%, and 9.5% of infection, respectively. Comparing our findings with the infection rates found previously in small mammals with molecular techniques, they are similar to those found in Chile [19, 27, 61] and in other countries [3, 62, 63]; however, they are quite different to previous reports from Bolivia and Argentina [64, 65].

As mentioned, the infection status of small mammals was explained by the host species, locality and study period. It is possible that the particular geo-climatic conditions–temperature, precipitation and elevation–could influence *T*. *cruzi* transmission among vectors and mammals, as reported in mice inoculated with *T*. *cruzi* isolates from higher elevation, which showed the lowest parasitemia [66]. However, differential availability of vertebrates could also explain these differences in small mammals’ infection between localities, since the localities with higher odds of infection for mammals presented also lower numbers of mammals captured. It is possible that when there are fewer individuals available, their probability of becoming the vectors’ blood-meal, and therefore, their chance of becoming infected, increase. Regarding the study period, during winter mammals showed lower odds of infection than during summer. Hosts seem to control infection after the acute period, reducing their parasitemia [24, 67]; therefore, this control could occur during the winter in our study system.

In triatomines we detected that *T*. *cruzi* infection rate was higher in *M*. *spinolai* than *T*. *infestans*. *Mepraia spinolai* has been described as the most relevant vector in the sylvatic transmission cycle of *T*. *cruzi* in Chile [68]; however, *T*. *infestans* has been traditionally considered the vector to humans [37], given its domestic and peridomestic habitat preferences and higher transmission efficiency by a faster post-feeding defecation than *M*. *spinolai* [69]. Although the lower *T*. *cruzi* infection detected in *T*. *infestans* could be an optimistic result, *T*. *cruzi* infection modifies *M*. *spinolai*’s behavior, reducing its defecation time [70], improving its ability as vector. Thus, *M*. *spinolai*’s relevance should not be neglected, considering its high infection rates and widespread distribution in North-Central Chile. Previous studies in sylvatic areas of Chile showed variable *T*. *cruzi* infection rates, ranging from 36.5% to 57.7% in *T*. *infestans* and 29.9%-76.1% in *M*. *spinolai* [32, 33, 36–38, 71]. Small mammal species composition at the localities where *T*. *infestans* and *M*. *spinolai* were found was slightly different, but their availability of larger mammals was probably very different. This may be explaining why in Locality 4, where both triatomine species were present, they showed similar infection rates, so different mammals’ availability may influence triatomines’ infection [37, 61]. Unfortunately, our design precluded the study of larger mammals.

Triatomine species was the only variable retained as predictor of triatomine infection status. Locality and study period seemed to be less relevant for triatomines’ infection status than to hosts. Previous studies have shown temporal variations in density and infection rates of hosts and vectors, comparing different years [33, 68, 72]. Here we analyzed two study periods within one year, and small mammals were significantly more infected during summer than winter. On the contrary, triatomines’ infection was higher during winter than summer. Previous studies have shown that abundance and home range of hosts and triatomines increase during summer in the North-Central Chile and Argentina [22, 57, 73–75]. Additionally, the maximum overall densities of *M*. *spinolai* occurred in summer months [76]; accordingly, a study of *T*. *infestans* in Argentina showed higher densities in houses between spring and autumn and a decrease in winter, and also that the number of parasites in triatomines’ rectal contents showed seasonal changes, with higher values in late spring [77]. In *Triatoma protracta*, lower environmental temperatures retarded and higher temperatures increased the number of metacyclic trypomastigotes released in its dejections [78]. During warm weather there was a larger diversity of alimentary sources than in cold weather in *M*. *spinolai* [79], supporting the idea of higher *T*. *cruzi* transmission risk to small mammals during warmer months in Chile that could have led to high *T*. *cruzi*’s parasitemia and higher detection of infection in their blood samples.

Vector population composition varied between study periods for *M*. *spinolai*, with concomitant higher infection rates in winter [80]. Also, it is possible that infection in triatomines is more easily detected after some time since parasite ingestion, allowing *T*. *cruzi* to multiply [81]. Sylvatic *Triatoma brasiliensis* showed higher infection when its nutritional status was better [82]. Long fasting periods can eliminate 99.5% of *T*. *cruzi* flagellates in the triatomines’ rectum [83], explaining why *M*. *spinolai* increased its infection rate in dejections with supplementary feeding [84]. We expected that during winter the availability of hosts were lower, but we captured lower numbers of small mammals during winter only in two localities, so this may suggest that triatomines captured during winter could have maintained their *T*. *cruzi* populations. Unfortunately, nutritional status of the captured triatomines was not evaluated.

In this study, the number of triatomines caught during summer was almost six times the number of triatomines found during winter; it is possible that a differential bait attraction could account for this difference. However, previous studies have shown that *M*. *spinolai* and *T*. *infestans* are not active when temperature is below 15 ºC [35], which is frequent during the cold season in the sampled localities. The higher infection rates detected in winter may have been related to this lower sample size, but in laboratory, *T*. *cruzi*-infected *M*. *spinolai* showed reduced time to detect potential hosts in comparison to uninfected insects [70], so they might have been able to find baited traps more easily in adverse climatic conditions.

A high number of positive samples did not hybridize with any of the probes used in our study. It is possible that some samples corresponded to DTUs not tested in this study (TcIII or TcIV) or to the genotype TcBat. It is also possible that the unidentified samples corresponded to one of the tested DTUs, but with slight genetic differences that prevented hybridization, as reported in other endemic areas [85]. This is particularly relevant for TcI, with greater internal diversity than the other DTUs [86]. Another possibility explaining our low efficiency in the hybridization tests is a reduced amount of kDNA transferred to the nylon membranes.

TcI was the most frequently detected DTU in small mammals and triatomines, agreeing with previous reports in small mammals [16, 25, 27], triatomines [36, 37, 51, 87], and humans in Chile [17, 51], and in sylvatic and domestic cycles of America [88]. Regarding the type of infection, this study agrees with others where single infections were more common than mixed ones in triatomines and small mammals [25, 27, 36, 37]. However, given our low number of positive samples effectively hybridized, we cannot be confident that this tendency would have remained the same under complete DTUs detection. We were able to detect mixed infections in four small mammal species. In triatomines, we detected higher number of mixed infections in *T*. *infestans*.

We found more infection clusters in triatomines than in small mammals. We also found clustering when combining triatomines and small mammals, agreeing with a report relating host probability of infection with their distance to *M*. *spinolai* colonies [38]. As supported here, there are spots in space where infected individuals aggregate. *Mepraia spinolai* exhibits a sit–and-wait strategy for finding hosts [22], and seems to feed on species according to availability [20, 22], same as *T*. *infestans* [79]. Therefore, triatomines’ cohorts from eggs laid in the same microsite would feed on the nearest available host. If these hosts had been infected, triatomines would become infected and later infect other small mammals, producing clustered spatial patterns of higher rates of infection. Moreover, infection among triatomines could be enhanced by coprophagy and cannibalism [4]. In mammals, potential congenital transmission [89, 90] could also perpetuate infection on site.

Locality 6 had most of the purely spatial clusters of infection, followed by Locality 5, where the ecotope providing shelter for both triatomines and small mammals were terrestrial bromeliads, presenting a spatially aggregated distribution [27, 32, 91]. Dispersion from these patches may be more difficult than from a continuous ecotope, as rocky outcrops, enhancing transmission in case they were infected. Future studies should evaluate the variables that differentiate cluster areas from the rest, which could be related with biotic conditions, as reported for sylvatic *Rhodnius spp*. inhabiting palms [92]. Locality 6 clusters were near human dwellings, so extra precautions should be taken to avoid exposure to vectors. Sporadic dwelling invasion of wild triatomines has been reported as the main vectorial risk in Chile [5].

We found purely temporal clusters of infection, with higher infection rates during summer in Localities 1 and 2. The temporal differences of infection in these localities might be related with the changes in density and composition of small mammals’ community, mainly due to differences in density and infection of the two most abundant small mammals, *Octodon* sp. and *P*. *darwini*.

The detected spatio-temporal clustering of infection shows sites presenting different rates between periods. Site’s microclimatic conditions may vary transmission, or individuals could aggregate in these sites during some periods.

In sum, our study evaluated *T*. *cruzi* infection, described DTUs and clustering from locations in a vast geographical extension during two contrasting seasons, determining in a widespread manner *T*. *cruzi* infection distribution in host and vector populations simultaneously, unveiling some of the eco-epidemiological complexity of *T*. *cruzi* wild cycle in Chile.

### Conclusion

This study describes *Trypanosoma cruzi* infection status, infecting DTUs, and determines the spatial and temporal variations of infection in small mammals and triatomines of the endemic zone of Chile. *Octodon sp*. and *Phyllotis darwini* were the most represented small mammals, and they showed high infection rates, thus representing important wild hosts. *Mepraia spinolai* presented higher infection rate than *Triatoma infestans*; however, non-domestic populations of both vectors were infected in all localities and study periods evaluated, emphasizing the need for sustaining prevention measures even if domestic vectorial transmission has been interrupted. We detected the four tested DTUs in triatomines and small mammals, with an overall predominance of TcI, following the trend of Chile and America. Significant spatial, temporal and spatio-temporal clusters for infection were detected within localities, mainly in triatomines. Finally, we can conclude that *T*. *cruzi* infection varies between host and vector species, localities and study periods in North-Central endemic zone of Chile.

## Supporting information

S1 Table Geographic and bioclimatic description of the study sites. (DOCX)

S2 Table *Trypanosoma cruzi* infection in small mammals and triatomines. Number of captured individuals by species, locality and study period. Percentage of infection with *T*. *cruzi* between parentheses.
(XLSX)

S3 Table Number of individuals with detected DTU by species, locality and study period. One individual might be infected with more than one DTU.
(XLSX)

S4 Table Description of statistically significant spatial, temporal or spatio-temporal clusters of infection status detected in small mammals, triatomines or both. (XLSX)

S1 Fig **A:** Rectangle showing the location of North-Central Chile in South America. B: Close up on the rectangle, showing the six localities prospected in North-Central Chile. Geographical coordinates, WGS84.
(TIF)
