# Supplementary material for: Spatio-temporal characterization of Trypanosoma cruzi infection and discrete typing units infecting hosts and vectors from non-domestic foci of Chile
Source: PLoS Negl Trop Dis. 2019 Feb 15;13(2):e0007170. doi: 10.1371/journal.pntd.0007170 (PMC6395009; doi:10.1371/journal.pntd.0007170)
Supplement: S1 Table — (DOCX) [file pntd.0007170.s001.docx]

| Locality number | Locality name | Coordinates (Lat - Long) | Mean±SD Elevation  (m.a.s.l.) | Mean Temperature (range) °C | Annual Precipitation (mm) | Climatic classification | Vegetation | Ecotope |
| --- | --- | --- | --- | --- | --- | --- | --- | --- |
| 1 | El Maqui | 30°49’S-70°39’W | 1083±22 | 12.1 (5.7-18.5) | 130 | BSk  (arid steppe, cold arid) | High Andean steppe with shrub steppe of Coquimbo | Rock piles and shrubs |
| 2 | Las Chinchillas National Reserve | 31°31’S-71°06’W | 507±14 | 14.3 (8.2-20.3) | 212 | BSk  (arid steppe, cold arid) | Interior steppe scrub | Rocky outcrops and shrubs |
| 3 | El Sobrante | 32°14’S-70°48’W | 886±21 | 15.2 (7.8-22.5) | 232 | BSk  (arid steppe, cold arid) | Thorn scrubs | Rock piles and shrubs |
| 4 | Putaendo | 32°36’S-70°40’W | 886±17 | 16.0 (8.0-24-0) | 276 | BSk  (arid steppe, cold arid) | Thorn scrubs | Rock piles and shrubs |
| 5 | Til-Til | 33°05’S-70°56’W | 718±34 | 14.2 (6.8-21.5) | 342 | Csc (warm temperate, with dry and cool summer) | Thorn scrubs | Terrestrial bromeliads of the genus *Puya* |
| 6 | Calera de Tango | 33°39’S-70°47’W | 535±13 | 15.2 (8.0-22.3) | 405 | Csc (warm temperate, with dry and cool summer) | Spiny scrubs of the coastal mountain range | Terrestrial bromeliads of the genus *Puya* |
