# Supplementary figures and images for: Spatio-temporal characterization of Trypanosoma cruzi infection and discrete typing units infecting hosts and vectors from non-domestic foci of Chile
Source: PLoS Negl Trop Dis. 2019 Feb 15;13(2):e0007170. doi: 10.1371/journal.pntd.0007170 (PMC6395009; doi:10.1371/journal.pntd.0007170)

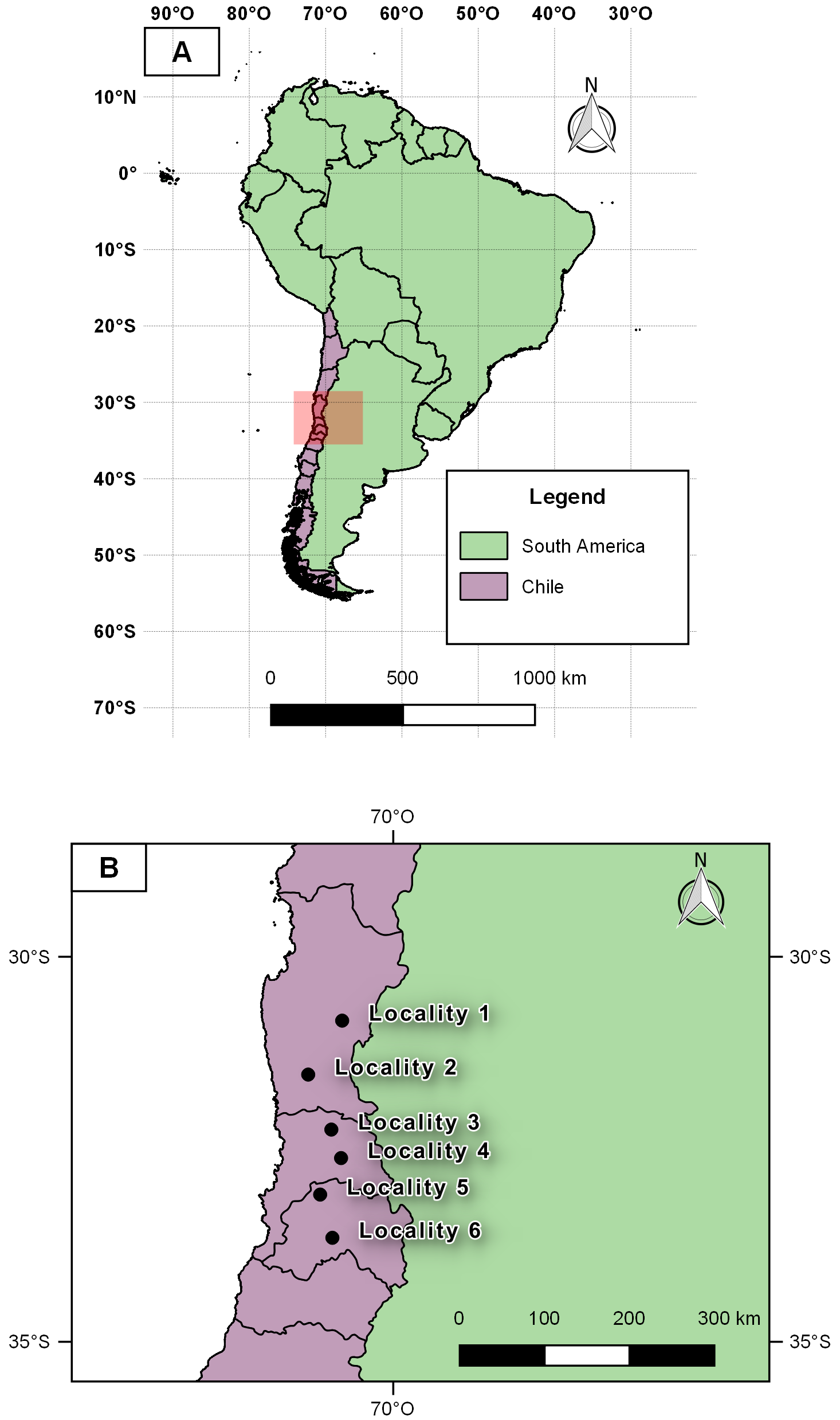

Supplement: S1 Fig — A: Rectangle showing the location of North-Central Chile in South America. B: Close up on the rectangle, showing the six localities prospected in North-Central Chile. Geographical coordinates, WGS84. (TIF) [file pntd.0007170.s005.tif]
